# Gastric polyp detection in gastroscopic images using deep neural network

**DOI:** 10.1371/journal.pone.0250632

**Published:** 2021-04-28

**Authors:** Chanting Cao, Ruilin Wang, Yao Yu, Hui zhang, Ying Yu, Changyin Sun

**Affiliations:** 1 Beijing Engineering Research Center of Industrial Spectrum Imaging, School of Automation and Electrical Engineering, University of Science and Technology Beijing, Beijing, China; 2 Institute of Automation, Chinese Academy of Sciences, Beijing, China; 3 Beijing An Zhen Hospital, Beijing, China; 4 School of Automation, Southeast University, Nanjing, China; Polytechnical Universidad de Madrid, SPAIN

## Abstract

This paper presents the research results of detecting gastric polyps with deep learning object detection method in gastroscopic images. Gastric polyps have various sizes. The difficulty of polyp detection is that small polyps are difficult to detect from the background. We propose a feature extraction and fusion module and combine it with the YOLOv3 network to form our network. This method performs better than other methods in the detection of small polyps because it can fuse the semantic information of high-level feature maps with low-level feature maps to help small polyps detection. In this work, we use a dataset of gastric polyps created by ourselves, containing 1433 training images and 508 validation images. We train and validate our network on our dataset. In comparison with other methods of polyps detection, our method has a significant improvement in precision, recall rate, F1, and F2 score. The precision, recall rate, F1 score, and F2 score of our method can achieve 91.6%, 86.2%, 88.8%, and 87.2%.

## 1. Introduction

Gastric cancer is one of the most common malignant tumors threatening human health [1, 2]. According to the statistics of the International Agency for Research on Cancer in 2012, there are about 951,000 newly diagnosed gastric cancer patients worldwide every year. The gastric polyp is a benign lesion with localized protuberance of gastric mucosal epithelium. The incidence of gastric polyps is 0.8-2.3%. The gastric polyp is divided into multiple and single polyps. According to pathological types, the gastric polyps can be divided into the adenomatous polyps and the non-adenomatous polyps. If the adenomatous gastric polyp is not treated in time, it will recur in the long-term and gradually become cancer. The adenomatous gastric polyp has a canceration rate of 10-20%, which is considered to be precancerous lesions. The canceration rate of gastric polyps increases with the increase of size. And the number of gastric polyps in the stomach also affects the canceration rate. Therefore, accurate detection of gastric polyps is very important.

Currently, the main way to check gastric polyps is gastroscope [3, 4]. The gastroscopic examination can directly observe the true condition of the site. However, it is difficult to accurately identify gastric polyps from the gastroscope. There are mucous membranes on the gastric wall, and the mucous membranes will form a large number of folds. Some of these folds are similar to gastric polyps, making polyps difficult to recognize. Some gastric polyps are small in shape. They are very easy to be missed or misdiagnosed. Accurate diagnosis of gastric polyps through gastroscopy is a challenge for doctors. Even in the process of specialist examination, there will be a certain rate of gastric polyps missed detection. Some gastric polyps even need experts to diagnose accurately, which will lead to untreated in time.

**Fig 1:**
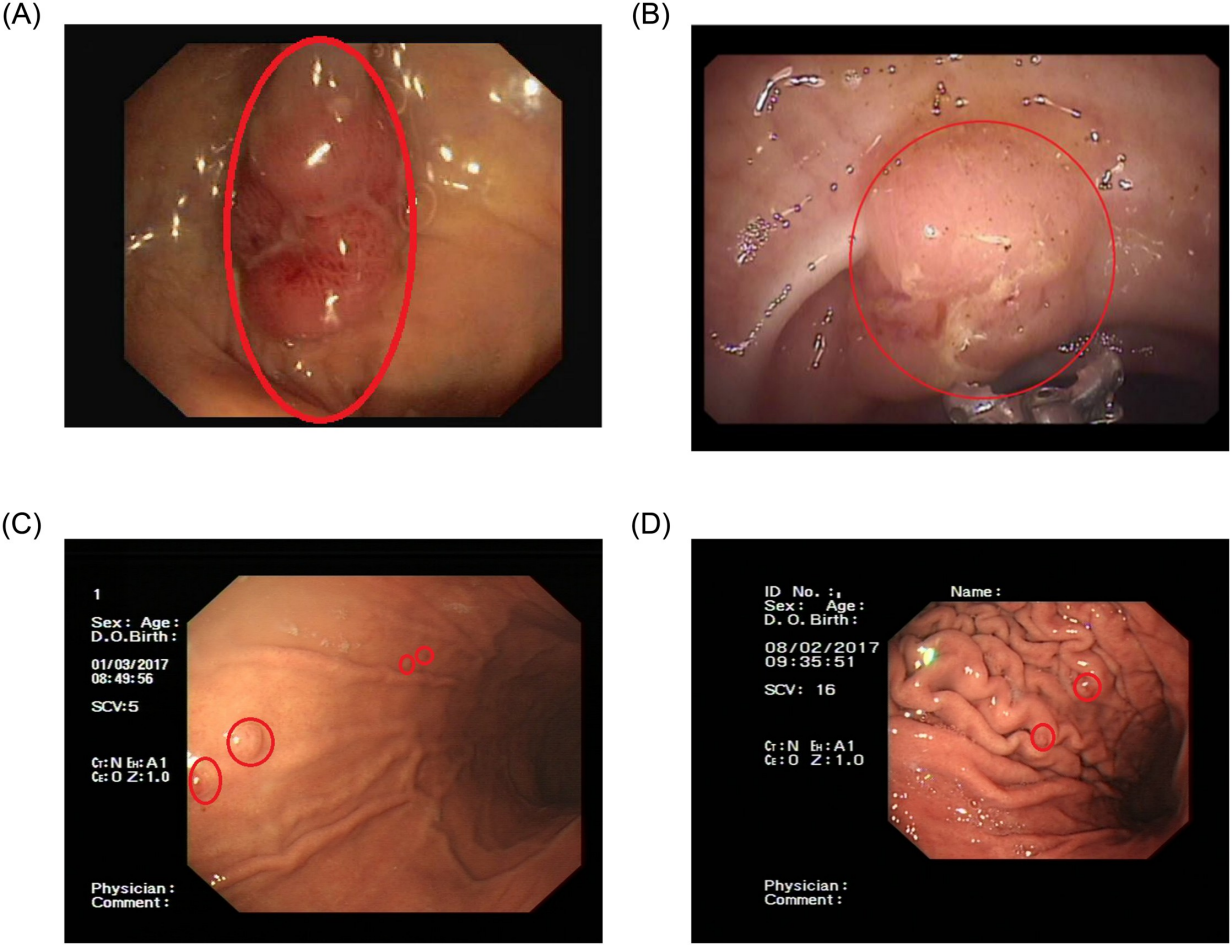
Comparison of colonic polyps and gastric polyps. The first two images ((A) and (B)) are images of colonic polyps. The next two images ((C) and (D)) are images of gastric polyps.

To solve these problems, many computer-aided detection methods based on the traditional methods [5–7] are applied in the detection of polyps. However, if the polyps features are not obvious or have a lot of interference in the images, these traditional methods can not be accurately detected polyps. Therefore, traditional methods have poor detection performance in polyps detection. Deep learning methods [8–10] have strong feature extraction capability, which has achieved great results in the field of general image processing. Meanwhile, these methods have rapid development in the field of medical image processing [11, 12]. Currently, [13] had applied Faster R-CNN without the sub-network to detect colonic polyps. [14] had applied YOLO with an Efficient Convolution Operators(ECO) tracker to detect colonic polyps. [15] had applied the YOLO network to detect colonic polyps in real-time. [16] had used a region-based CNN for the automatic detection of colonic polyps in the images and videos. [17] had used a real-time object detector based on YOLO to detect colonic polyps. There are also some other colonic polyps detection methods based on deep learning [18–21]. The gastric polyps in our dataset are different from the colonic polyps in the pubic datasets. On the one hand, most of the polyps in the common colonic polyps datasets not only are prominent but also located in the middle of the image. Most of a colonic image contains only one colonic polyp [22–24]. However, our dataset contains both large gastric polyps and small gastric polyps, and most of them are small gastric polyps. And most of the images in our dataset contain more than two gastric polyps. On the other hand, the background of the intestine is simpler than that of the stomach [22–24]. There is gastric mucosa on the stomach, and there are many folds on the mucosa [25]. Therefore, it is harder to distinguish the features of polyps from the background. As shown in Fig 1, colonic polyps are easier to detect and recognize. Most of the polyps detection work at present is colonic polyps detection [26, 27]. Due to the gastric folds on the gastric wall and the small size of the gastric polyps, the detection method of colonic polyps cannot be applied to gastric polyps detection in our dataset.

At present, deep learning has made new progress in the field of object detection methods. Object detection methods can be divided into two main categories in the general image detection. One popular frame is two-stage detectors [28–31]. Another popular frame is single-stage detectors, such as SSD [32], YOLO [33], YOLO9000 [34], etc. The ability of these methods to detect small objects is insufficient. The size of small objects in the original image is relatively small. In the general object detection model, the network predicts the object on the deep feature maps after several downsampling. However, in the deep feature maps of the detection network, the lack of detailed texture information required for small object detection results in poor detection of small objects. There are many small gastric polyps in our dataset. These methods can not accurately detect gastric polyps because of these small gastric polyps. Many special object detection methods [35–37] are proposed to detect small objects. These methods perform well in dealing with small objects. But they do not perform well in detecting objects of other sizes.

In this paper, to tackle these problems mentioned above, we propose a feature fusion and extraction module and combine this module with the YOLOv3 network [38]. In the traditional feature pyramid, the new features can only fuse features from the corresponding level features on the backbone network and higher levels [39]. And each fusion operation will dilute the feature information of non-adjacent levels in the process of feature information transmission. Compared to the traditional feature pyramid, our feature fusion and extraction module can fuse different level features at once and generate a new feature pyramid from the fused features, which can retain the features information of all levels. The feature fusion and extraction module deepens the network and obtains more semantic feature information, which helps to distinguish gastric polyps from gastric folds. And this module fuses the low-level features with the high-level features to improve the performance of gastric polyps detection. On the basis of the YOLOv3 network, our network further improves the ability of object detection, especially the ability of small object detection. In our gastric polyps dataset, each evaluation metric is increased by 3% to 5%. The key contributions of our work are summarized as follows:

- A feature fusion and extraction module. We propose a new feature fusion and extraction module that is designed to improve gastric polyps detection results. We combine the feature maps from different levels at once and generate a feature pyramid to fully utilize the features.
- The feature fusion and extraction module combined with the YOLOv3 network. We combine the feature fusion and extraction module with the YOLOv3 network to detect gastric polyps in our dataset. The whole network detection ability has been further improved. The precision, recall rate, F1 score, and F2 score of our network can achieve 91.6%, 86.2%, 88.8%, and 87.2% in our gastric polyps dataset.

The remaining sections are arranged as follows. Section 2 gives the structure for our network and the method for gastric polyps detection. Section 3 introduces the detail of our dataset and the public datasets. Section 4 introduces some details of the training process and experiment results. Section 5 discusses the results of gastric polyps detection. At last, Section 6 gives the conclusion of this work.

## 2. Proposed method

**Fig 2:**
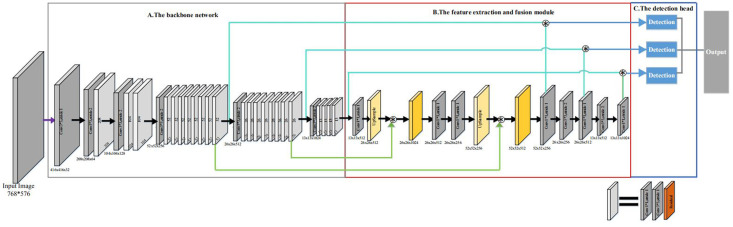
Architecture of our network.

In this work, we combine the feature extraction and fusion module with the YOLOv3 network to form a deep learning object detection network, which is shown in Fig 2. Our network consists of three modules: the backbone network, the feature extraction and fusion module, and the detection head. The backbone is used to extract and learning the image features. The detection head learns to detect objects from image features, such as box coordinates, categories, etc. We propose the feature extraction and fusion module to further feature extraction and better fusion of low-level features and high-level features. In the following, we describe each module of our network for gastric polyps detection in detail.

### 2.1. The feature extraction and fusion module

**Fig 3:**
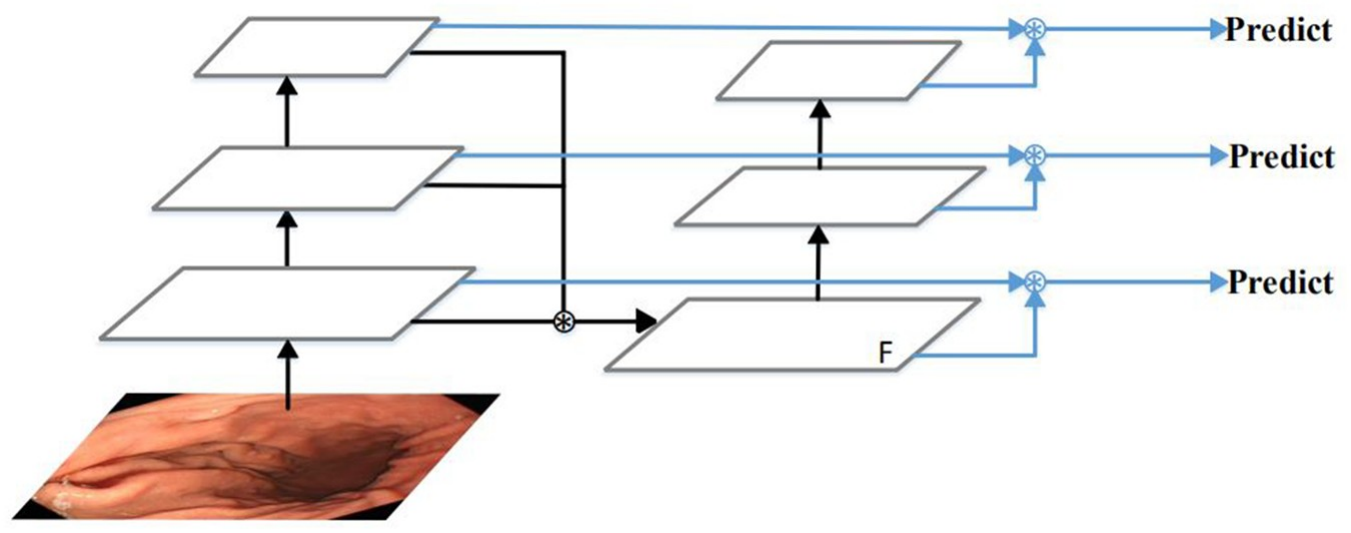
The feature extraction and fusion module.

Many algorithms try to observe and make full use of feature fusion to improve multi-scale object detection performance [40–46]. Among them, PAN performed very well [41]. Therefore, PAN structure is adopted in YOLOv4 [47]. However, PAN also has shortcomings, due to the false detection and missed detection of small objects. The reason is that the extraction of low-Level features lack the guidance of the highest-level of semantic information. Moreover, the design requires multiple feature fusion processes, which causes its processing speed to be very slow. Therefore, we propose a new feature extraction and fusion module, and its simplified structure is shown in Fig 3. The proposed feature extraction and fusion module fuse the different level features from the backbone network at once. The feature map F obtained contains both high-level semantic information and low-level detailed texture information, which is conducive to accurately identify and locate small polyps. This simple structure makes it faster than PAN. The detailed introduction of this module is as follows.

First of all, we fuse the features of the last three convolutional blocks at once from the backbone network using the bilinear interpolation and concatenation operation. The concatenation operation has more flexibility of fusing the feature maps and gets a better result than other ways. Then, we use several simple convolutional blocks to extract further semantic features. It is composed of a series of 3 * 3 and 1 * 1 convolutional layers. By inputting the fused feature maps into the convolutional blocks, we can not only deepen the depth of the convolution network but also ensure that the low-level features can be effectively utilized. Finally, we take the feature maps from the above convolutional blocks and merge them with the feature maps of the last three convolutional blocks from the backbone network using concatenation operation and several 1 * 1 convolutional layers to generate the feature pyramid. In the feature pyramid, we further fuse the low-level features with the high-level features. On the one hand, low-level features contain more details and image structure information. High-level features contain more semantic information. The fusion of low-level features and high-level features can improve the accuracy of object detection. On the other hand, in the feature pyramid network, large objects are predicted on deep feature maps. However, if the feature map is deepened, the sharpness of the object’s edge will become worse. It is difficult to accurately regress the boundary of an object. The resolution of the feature map is reduced to 1/32 of the original, or even smaller [48], where small objects are invisible. The fusion of low-level features and high-level features can guarantee that the information of small objects is not lost and large objects are detected accurately. Therefore, we fuse the low-level features with the high-level features in our network.

The feature fusion part of the last three convolutional blocks from the backbone network, these simple convolutional blocks, and the feature pyramid constitute the whole feature extraction and fusion module in our network, which has 36 layers. Based on guaranteeing the detection performance in YOLOv3 network, we combine this module with the YOLOv3 network. In our dataset, the performance of our method has improved significantly by using this module.

### 2.2. The backbone network

In our gastric polyps dataset, it is difficult to recognize polyps and background accurately. In order to extract features of gastric polyps accurately, the backbone network must have strong feature extraction ability. In our network, the backbone network is the Darknet-53.

The Darknet-53 has 74 layers, of which 53 convolutional layers. It is composed of a series of 3 * 3 and 1 * 1 convolutional layers and some residual connections. These residual connections can address vanishing gradient and exploding gradient problem. Compared to the other classification network(e.g. VGG16, ResNet-50, ResNet-101 and DenseNet), the Darknet-53 has stronger feature extraction ability. In the open ImageNet classification challenge, the top-1 accuracy rate of Darknet-53 is 77.2% and the top-5 accuracy rate is 93.8%. In this work, according to the resolution of the feature map after the residual connection, we divide the whole backbone network into six convolutional blocks, as shown in Fig 2.

### 2.3. The detection head

Our network makes use of three different scale detectors in the detection head. These three detectors detect small, medium, and large objects respectively. By using these three different scales of detectors, our network can better detect gastric polyps of different sizes. Every detector is composed of a series of 3 * 3 and 1 * 1 convolutional layers, which has 7 convolutional layers.

Our network predicts five offsets, *t*_*x*_, *t*_*y*_, *t*_*w*_, *t*_*h*_, *t*_*o*_, for bounding box. The first four are coordinates, and the last one is confidence. The whole gastric polyps image is divided into multiple cells during the detection process. (*c*_*x*_, *c*_*y*_) are the distance of the current cell and the top left corner of the whole image, *σ*(*t*_*x*_), *σ*(*t*_*y*_) are coordinate value of bounding box center and the top left corner of the cell, and *p*_*w*_; *p*_*h*_ are the preset length and width of anchors. After calculation, *b*_*x*_; *b*_*y*_; *b*_*w*_; *b*_*h*_ are the final predicting results of bounding box.

$$\begin{array}{c} b_{x}=\sigma \left(t_{x}\right)+c_{x} \end{array}$$ (1)

$$\begin{array}{c} b_{y}=\sigma \left(t_{y}\right)+c_{y} \end{array}$$ (2)

$$\begin{array}{c} b_{w}=p_{w}e^{t_{w}} \end{array}$$ (3)

$$\begin{array}{c} b_{h}=p_{h}e^{t_{h}} \end{array}$$ (4)

Then, our network uses non-maximum suppression (NMS) on the results obtained by these detectors to get the final detections.

### 2.4. Classification and detection

In this work, we complete stomach classification and gastric polyps detection tasks. Common public datasets such as ImageNet are very different from our dataset, the pre-training weights of training with common public datasets is not very suitable for our network. So we think about getting the pre-training weights of the backbone network by stomach classification tasks. Firstly, we train the classification network to get a well-trained backbone network and pre-training weights. Secondly, we train detection network by using pre-trained backbone network weights. This can accelerate the convergence of the detection network and reduce the detection network training time.

In order to complete the classification of different parts of the stomach, we add three different layers after the backbone network. These layers are the average pooling layer, the connected layer, and the softmax layer. By adding these layers, a standard classification network is formed. In our dataset, there are three different categories in total. Therefore, the softmax layer output size is three. After completing the stomach classification training, we retain the weight of the first 74 layers and remove the last three layers. In the training process of detection, our network loads this weight and then trains the complete detection network.

## 3. Dataset

We perform a total of two sets of experiments and first perform a comparison experiment on gastric polyp detection with advanced algorithms on a private gastric polyp dataset. To demonstrate the effectiveness of our algorithm, we further conduct a comparison experiment with the existing results on the public dataset of colonic polyps. Therefore, we use two datasets, which are the private gastric polyps dataset and the public colonic polyps dataset. The details are as follows.

### 3.1. The gastric polyps datasets

**Fig 4:**
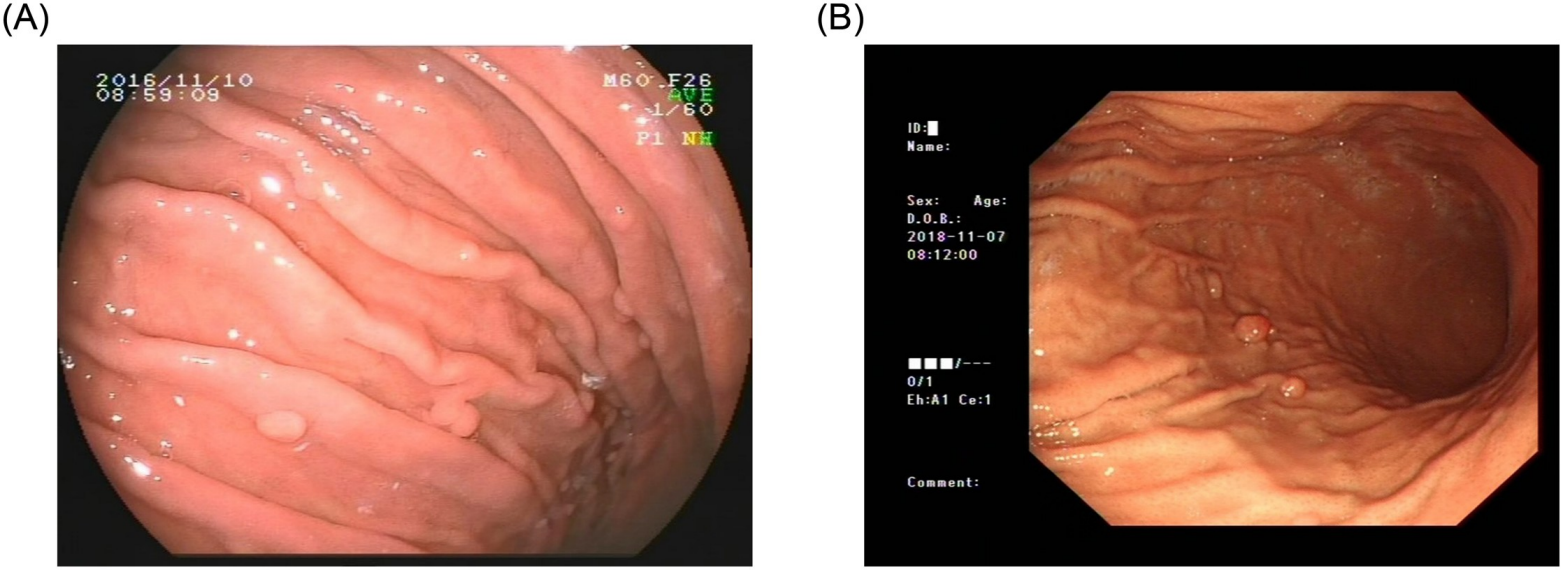
Two types of gastrointestinal polyps images using in this work. (A) Fujinon Iamge; (B) Olympus Image.

The gastric polyps image of our dataset was taken by two different devices, which are Fujinon and Olympus. As shown in Fig 4, these two devices are different in the final shooting results. We collected images from 406 patients undergoing endoscopy at Beijing Anzhen Hospital, Capital Medical University from January 2017 to December 2018. Among them, there were 1941 images with polyps and 329 images without polyps. A total of 2270 images. We were authorized to anonymously obtain gastroscopy images, which were collected from Beijing Anzhen Hospital, Capital Medical University. All the patients provided written informed consent for their medical images to be published and used in this research. Our study was approved by the Ethics Committee of Beijing Anzhen Hospital, Capital Medical University. The ethics approval number was 2020064X. In the stomach classification task, we only needed to label the category of each image. However, the detection task needed to know the exact location of the polyps in each image. Therefore, we divided the dataset into a classification version and a detection version. Each image was labeled by professional physicians to ensure that the labels of polyps don’t go wrong.

In this work, there are totally 2270 images of different gastric polyps patients, which are categorized into three main categories(upper stomach, middle stomach, and lower stomach). There are a total of 1589 images in the training set, including 577 images of the upper stomach, 718 images of the middle stomach, and 294 images of the lower stomach, While validation set has 681 images, which includes 247 images of the upper stomach, 308 images of middle stomach and 126 images of lower stomach, as shown in Table 1.

**Table 1:** Classification of the training image set and validation image set.

Category	Training set	Validation set
Upper stomach	577(36.3%)	247(36.3%)
Middle stomach	718(45.2%)	308(45.2%)
Lower stomach	294(18.5%)	126(18.5%)
Total	1589(100%)	681(100%)

In the gastric polyps detection work, the dataset of gastric polyps detection only includes 1941 images with polyps. The training set includes 1433 images, while the validation set includes 508 images. Each image is labeled by a professional physician. Compared to those public datasets, most of the gastric polyps are smaller and more numerous in our dataset.

### 3.2. The public colonic datasets

The most widely used public dataset is CVC-ClinicDB [49], which aims to detect colon polyps. This public dataset mainly includes 612 polyps images. Some other similar public datasets include ETIS-Larib Polyp DB [50] and ASU-Mayo Clinic Colonoscopy Video (c) Database [51]. These public datasets are all about colon polyps detection and colon polyps segmentation. Another public dataset for gastrointestinal diseases is the Kvasir dataset [52].

## 4. Experiments

In the introduction to the dataset, we mentioned that this work includes two sets of experiments, namely: a comparison experiment with advanced algorithms for gastric polyp detection, and a comparison experiment with existing results for colon polyp detection.

### 4.1. Evaluation metrics

Here are a few common model evaluation terms. When the predicted box falls into the ground truth area, the result is considered as True Positive (TP). On the contrary, it is False Positive (FP). When no predicted boxes were given on images of no ground truth area, the result is considered as True Negative (TN). On the contrary, it is False Negative (FN). Precision is also known as Sensitivity or True Positive Rate (TPR), which is the proportion of true positive in the identified images. The recall rate represents the proportion of all true positive in the detection dataset that were correctly identified as true positive. F1 score and F2 score are measures of detection’s accuracy, which consider precision and recall rate at the same time. F1 score considers that precision and recall rate are the same important. Compared to the F1 score, the F2 score considers that the recall rate is more important than precision. Thus, we can calculate precision, recall rate, F1 score, and F2 score by the following formulas:

$$\begin{array}{ccc} Precision & = & \frac{TP}{TP+FP} \end{array}$$ (5)

$$\begin{array}{ccc} Recall & = & \frac{TP}{TP+FN} \end{array}$$ (6)

$$\begin{array}{ccc} F1\;score & = & \frac{2\times Precision\times Recall}{Precision+Recall} \end{array}$$ (7)

$$\begin{array}{ccc} F2\;score & = & \frac{5\times Precision\times Recall}{4\times Precision+Recall} \end{array}$$ (8)

### 4.2. Implementation details

In this work, stomach classification is the first step. We use a classification network based on the Darknet-53 network as a training network for stomach classification. The method of weight initialization uses random initialization, rather than pretraining weights. This is because the datasets used for pre-training weights are very different from our dataset. Pre-training weights using the public datasets have little effect on our final training results. Thus, we choose to train the whole network from scratch.

Compared to those large public datasets, our dataset is so small. Therefore, we extend our dataset by random data augmentation methods during the training process. Random data augmentation is the random processing of data in each batch. The main random data augmentation methods are as follows: image rotation, image hue change, image saturation, and exposure change, etc. The angle of the image rotates from -10 degrees to +10 degrees. The hue of the image varies from -0.1 to +0.1. The saturation, exposure, and aspect of the image vary from 0.75 to 1 times. It also needs to consume a lot of system resources while augmenting data.

In the process of stomach classification, the initial learning rate is 0.001, momentum is 0.9, and weight decay is 0.0005. Learning rate adjustment strategy includes constant, steps, exp, poly, step, sig, and random. Poly strategy is used in this work. SGD is used in this work. This network is trained with CUDA9.0 and cuDNN backends. The GTX1080Ti is used in our training process. The batch size is 128 and the subdivisions are 16 in this work.

Then, we train the whole detection network to detect gastric polyps. We use the previous 74 layers of trained weights of the classification network as pretraining weights to train this network. The type of the network parameters is similar to that of the previous stomach classification training process. In the process of training detection network, the initial learning rate is 0.001, momentum is 0.9, and weight decay is 0.0005. The angle of the image rotates from -15 degrees to +15 degrees. The hue of the image varies from -0.1 to +0.1. The saturation, exposure, and aspect of the image vary from 1 to 1.5 times. The jitter of the image is 0.3, which can achieve data augmentation through jitter. Learning rate adjustment strategy is steps. In the detection process, ignore thresh is 0.5. When the IOU of the prediction bounding box and ground truth more than the ignore thresh, this bounding box is used to calculate the loss function. The other parameters are set the same as the classification network. We train the YOLOv3 network, our network and our network without the feature extraction and fusion module on our dataset. The other two networks provide a comparison for our network.

### 4.3. Experiment results

#### 4.3.1. Stomach classification and gastric polyp detection

Firstly, the results of the stomach classification need to be validated. The results of stomach classification include judging which part of the stomach belongs to and pointing out the probability of belonging to each part. In this work, the validation set of stomach is divided into 3 categories, 681 images. The classification results of the upper stomach, middle stomach, and lower stomach are 75.3%, 73.7%, and 73.1%, which are summarized in Table 2. When the result of a certain category is greater than 50%, we think it is recognized as this category.

**Table 2:** Results for stomach classification.

	Total	Correct classification	Error classification	Accuracy
Upper stomach	247	186	61	75.3%
Middle stomach	308	227	81	73.7%
Lower stomach	126	92	34	73.1%

**Fig 5:**
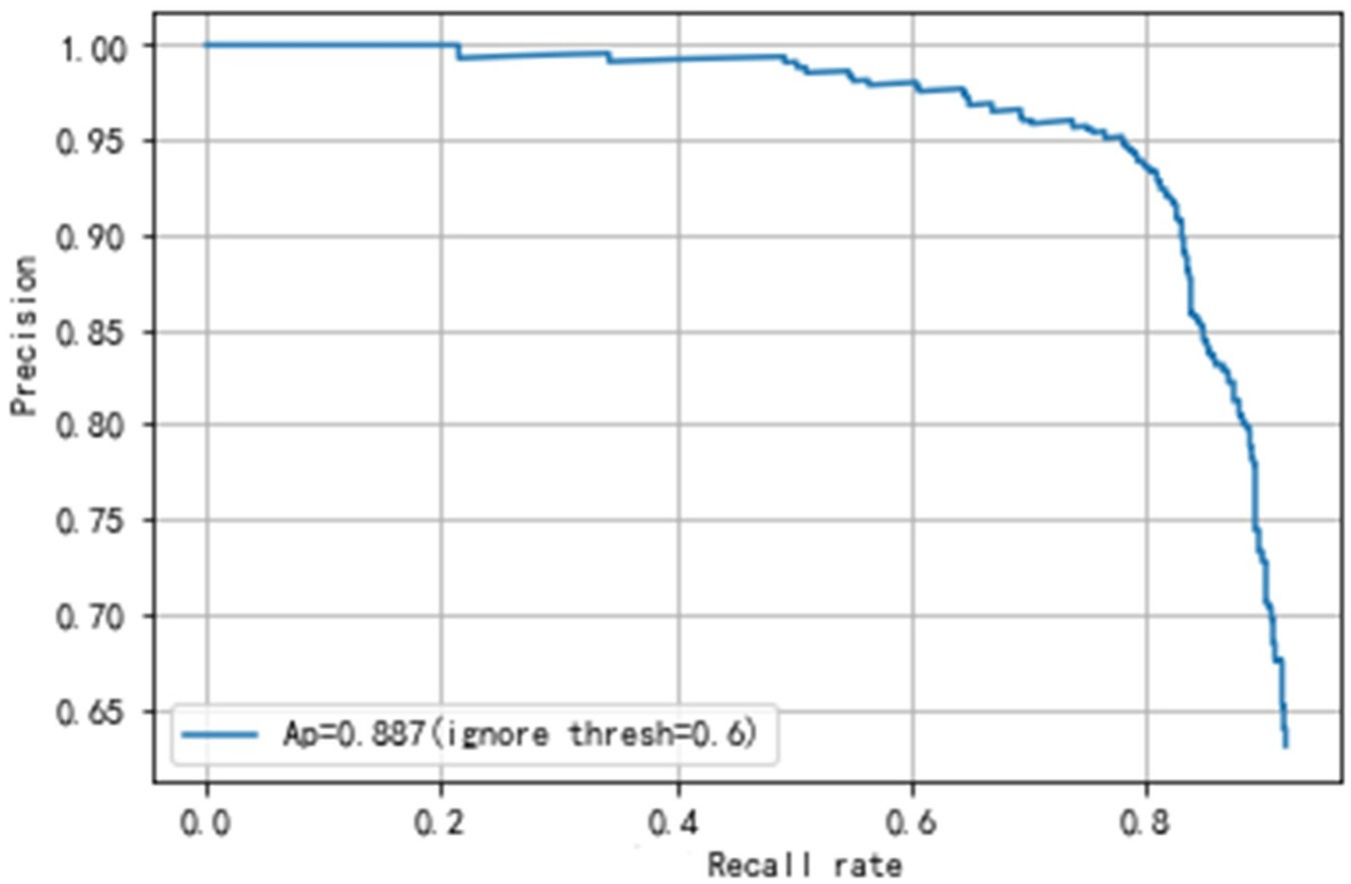
The PR curve of YOLOv3 and our network.

**Fig 6:**
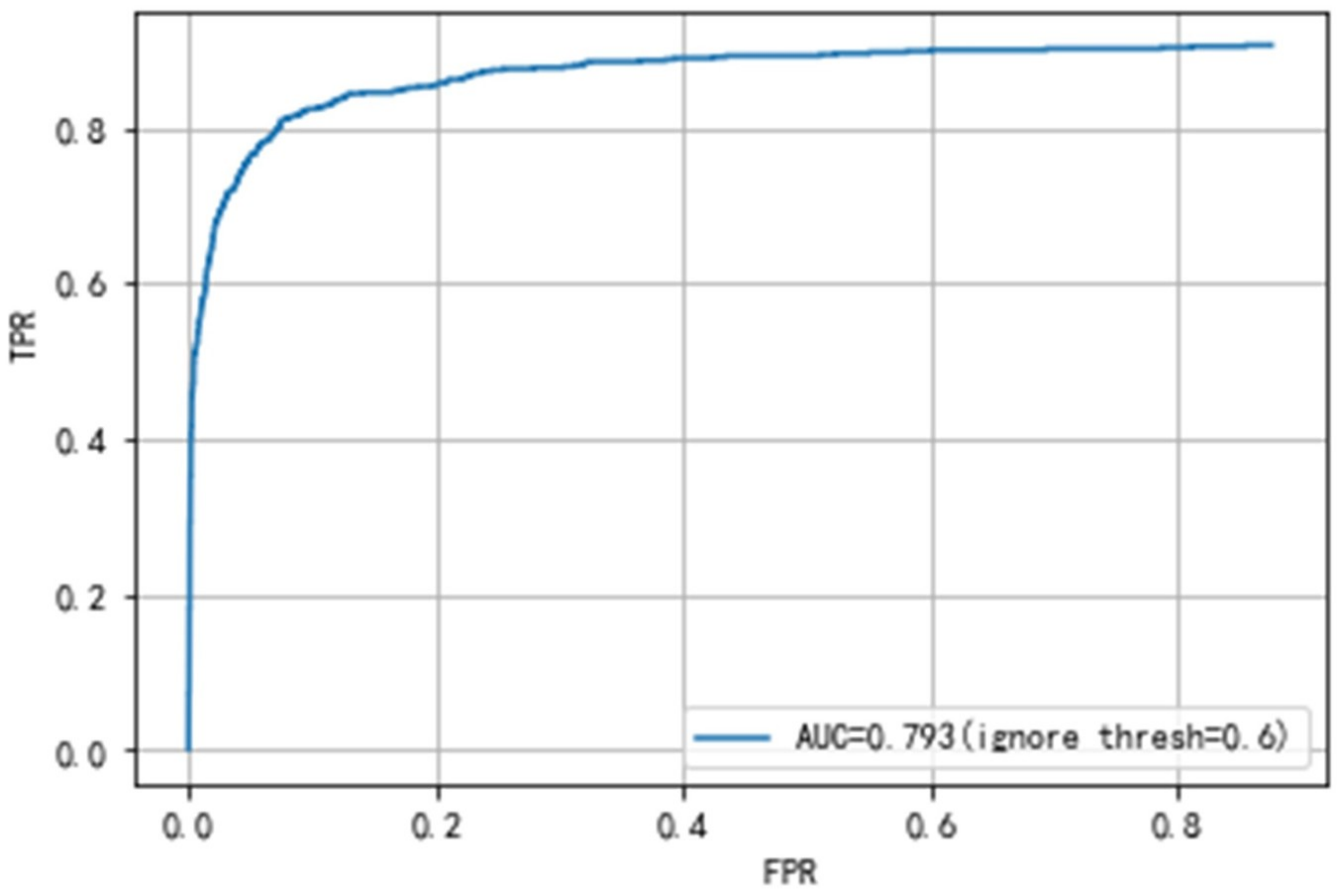
The ROC curve of YOLOv3 and our network.

Secondly, the results of the gastric polyps detection need to be validated. The gastric polyps detection task includes predicting whether an image has polyps and localizing the exact location of polyps. The detection validation set of our dataset contains 508 images, 661 polyps. We get the detection results of each image through the network, and then compare the results with the ground truth by manual labeling. Meanwhile, we calculate the mean Average Precision(mAP) of the final detection results to judge the performance of the network. We can also get TP, FP, TN, FN, and then calculate the precision, recall rate, etc. Finally, our proposed network achieves an Average precision of 88.7% and an AUC value of 79.3%, as shown in Figs 5 and 6. Our final results’ precision, recall rate, F1 and F2 score are 91.6%, 86.2%, 88.8% and 87.2%, as shown in Table 3.

**Table 3:** Summary of results for gastric polyps detection.

	TP	FP	FN	Precision[%]	Recall[%]	F1[%]	F2[%]
Faster R-CNN	535	72	126	88.1	80.9	84.4	82.2
SSD	535	160	126	77.0	80.9	78.9	80.1
YOLOv3	550	90	111	85.9	83.2	84.5	83.7
YOLOv3+PAN	565	77	96	88.0	85.5	86.7	86.0
YOLOv3-spp	564	81	97	87.4	85.3	86.4	85.7
YOLOv4	565	48	96	92.2	85.5	88.7	86.7
Our network(without the feature extraction and fusion module)	41	11	620	78.8	6.2	11.5	7.6
Our network	570	52	91	91.6	86.2	88.8	87.2

In addition, to improve the validity of the experimental results, we use the 10-fold cross-validation method on YOLOv3, YOLOv4, and our proposed network. We divide the dataset into ten parts, and then take turns to experiment with nine parts as training data and one part as test data. The corresponding average precision (AP) values are obtained in each experiment. Finally, the average of these experimental results is used as the final value. The experimental results are shown in Table 4. The average precision obtained by our network is higher than that of YOLOv3 and YOLOv4. This shows that compared with YOLOv3 and YOLOv4, our network is more suitable for detecting gastric polyps.

**Table 4:** The average precision of different networks in 10-fold cross validation.

Training time	Average Precision of YOLOv3 [%]	Average Precision of YOLOv4 [%]	Average Precision of Our network [%]
1	82.7	88.9	88.6
2	81.3	89.3	89.7
3	82.4	88.5	90.5
4	81.6	89.4	89.3
5	83.5	88.2	88.7
6	82.3	87.3	90.4
7	81.8	89.6	87.2
8	83.5	88.1	89.1
9	80.7	89.8	88.5
10	82.1	87.3	89.2
average	82.2	88.6	89.1

#### 4.3.2. Colonic polyp detection

We apply our method to the task of colonic polyp detection. The results show that our method can not only obtain excellent performance in the detection of gastric polyps, but also for the detection of colonic polyps. We train and verify our method on the public colonic polyps datasets, and compare the performance evaluation results with the methods in some studies in recent years, as shown in Table 5. Our method performs better than other methods on the evaluation metric. Therefore, our method is more effective in detecting polyps. The main reasons for these results include stronger feature extraction ability and object detection ability of our network. Our method can better distinguish polyps from the background and mark them out.

**Table 5:** Comparison of performance for colonic polyps detection.

Method	Description	dataset	Precision[%]	Recall[%]	F1[%]
Y. Shin et al. [53]	Augmentation-I	CVC-CLINIC&ETIS-LARIB	86.5	80.3	83.3
	Augmentation-II	CVC-CLINIC&ETIS-LARIB	91.4	71.2	80
M. Liu et al. [54]	SSD-ResNet50	CVC-CLINIC&ETIS-LARIB	72.6	80.3	76.3
	SSD-InceptionV3	CVC-CLINIC&ETIS-LARIB	73.6	80.3	76.8
	SSD-VGG16	CVC-CLINIC&ETIS-LARIB	62.2	75.9	68.4
S. Sornapudi et al. [55]	Region-based	CVC-CLINIC&ETIS-LARIB	72.9	80.3	76.4
Deeba et al. [56]	Without enhancement	CVC-CLINIC&ETIS-LARIB	32.90	36.54	34.62
	WE-PCA-SVM	CVC-CLINIC&ETIS-LARIB	35.38	44.23	39.31
	WE-SVM	CVC-CLINIC&ETIS-LARIB	38.08	47.60	42.31
	WE-RB	CVC-CLINIC&ETIS-LARIB	41.37	55.23	47.31
	WE-PCA-RB	CVC-CLINIC&ETIS-LARIB	49.52	74.04	51.66
Proposed	Our network	CVC-CLINIC&ETIS-LARIB	92.6	87.9	90.2

## 5. Discussion

In the previous section, we present a deep learning method for detecting gastric polyps. We train and validate this method on our gastric polyps dataset. There are more small size gastric polyps in our dataset than small size colonic polyps in public colonic polyps datasets. The existence of these small polyps increases the difficulty of detection. If these small gastric polyps are not handled properly, the accuracy of detection will be greatly affected. And our dataset contains not only small polyps but also large polyps. It is also an important aspect to solve the problem of polyps detection at different scales. We propose the feature extraction and fusion module to improve the detection ability of small gastric polyps and feature extraction ability. Therefore, our network has solved these problems and achieved good results.

Comparison of the results with other state-of-the-art object detectors are shown in Table 3. These methods are trained and validated on our dataset. There is a key parameter, ignore thresh, in our method. Ignore thresh can influence the precision of gastric polyps detection. The higher the value of ignore thresh, the higher the precision, the lower the recall rate, vice versa. In our work, we finally choose the ignore threshold of 0.6. When the ignore threshold is 0.6, our method can not only achieve high precision but also achieve high recall rate.

**Fig 7:**
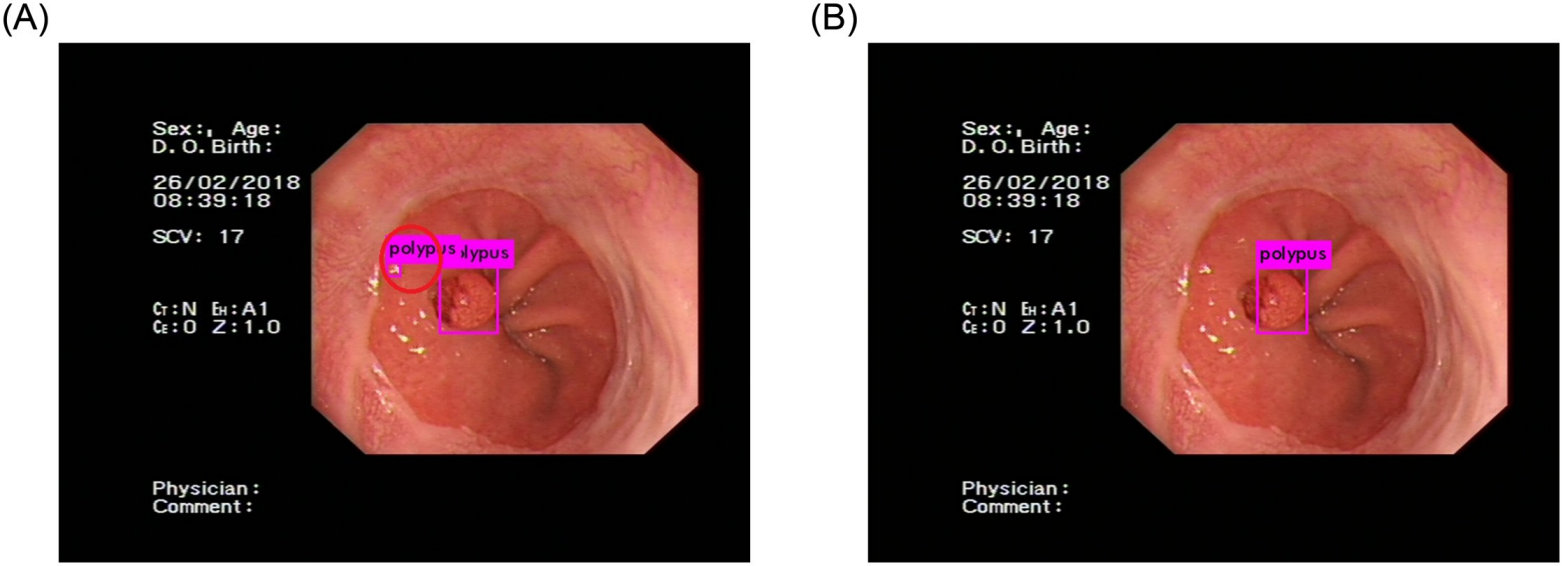
Polyp detection result. (A) is the detection result of the YOLOv3 network, which is incorrect. The background is considered gastric polyps. (B) is the detection result of our network, which is correct.

Compared with the YOLOv3 network, our network has better results. An important factor is that our dataset has many small gastric polyps, the YOLOv3 network does not have enough capacity to detect these polyps. When the number of detection network layers is deepened, some features of small gastric polyps will be lost, which will lead to a decrease in detection accuracy. Therefore, the recall rate of the YOLOv3 network is lower than our network. Another important factor is that reflective light will affect the final detection results when obtaining gastric polyps images. The shape of these reflective areas is similar to that of gastric polyps. If the feature extraction ability of the network is not strong, these areas will be considered polyps. When we use the YOLOv3 network for detection, this kind of error often occurs, as shown in Fig 7(A). Our network has solved this problem by using the feature extraction and fusion module. Compared to the YOLOv3 network, this module can extract and fuse more useful semantic features from each image. In the same image, the final detection result of our network is correct, as shown in Fig 7(B). Because there are many similar situations in gastroscopic images, the network must be able to accurately detect gastric polyps. Therefore, the precision of the YOLOv3 network is lower than our network.

**Fig 8:**
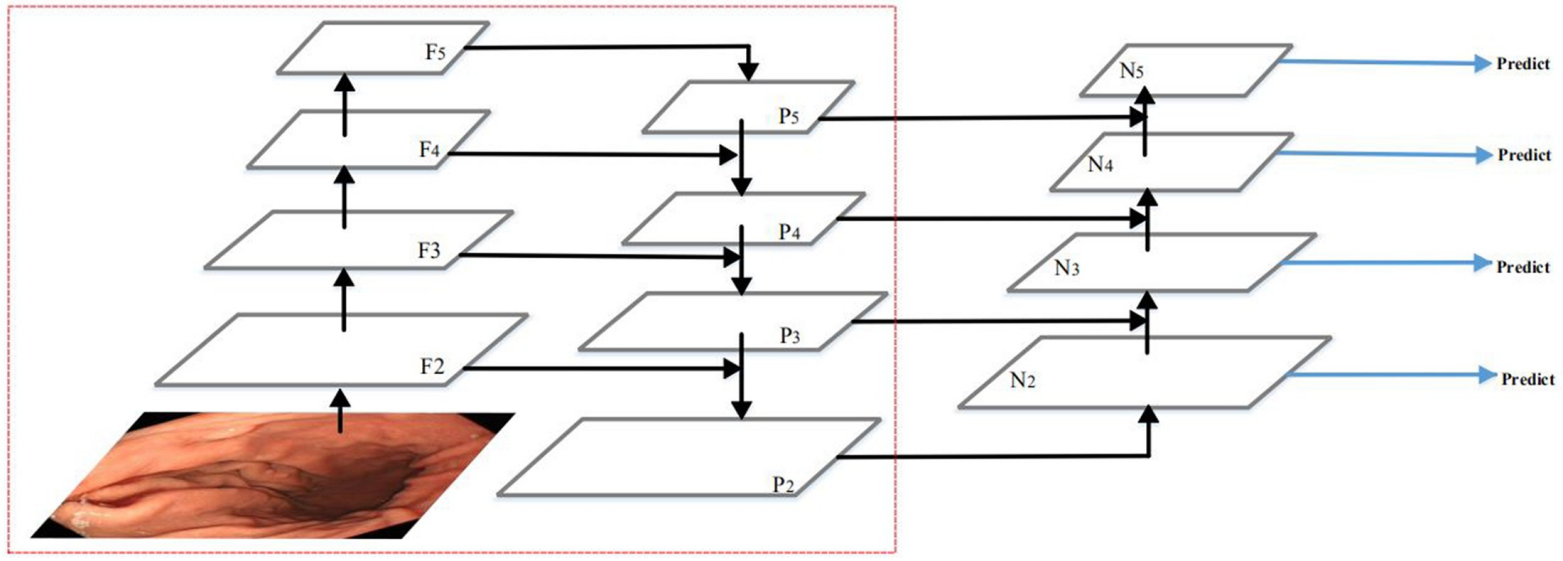
Structure of PAN.

We use PAN (Path Aggregation Network) [41] in YOLOv3 to form the detection network and evaluate its polyp detection results. The results show that our method is more effective for gastric polyp detection tasks, especially for the detection of small polyps. This is mainly due to the proposed feature extraction and fusion module. In PAN, the lower-level features lack the highest-level semantic information, resulting in some small polyps cannot be distinguished from the background, as shown in Fig 8. Moreover, our feature extraction and fusion module uses concatenation to merge different feature maps together, while PAN uses element-wise summation. Element-wise summation causes a lot of information loss, concatenation can get a better result than element-wise summation.

As shown in Table 3, the method proposed in this paper is better than YOLOv3-spp. YOLOv3-spp adds the spatial pyramid pooling module based on YOLOv3, which enriches the expressive ability of feature maps. It is suitable for complex multi-object detection. In our polyp detection task, its performance is worse than the YOLOv3 structure using PAN. The result of polyp detection with YOLOv3-spp is inferior to our method.

Compared with YOLOv4 [47], our method is lower than it in precision due to mistaken background for polyps. But the other three aspects are better than YOLOv4, because our method can effectively reduce the missed detection rate of small polyps. YOLOv4 adopts CSPDarknet53 as the backbone and modified the spatial attention module (SAM). The addition of the SAM helps distinguish background and polyps. In our method, the feature extraction and fusion modules are conducive to identifying small polyps. This enlightens us that we can further improve the performance of the network by adding the spatial attention module or improving the backbone network.

**Fig 9:**
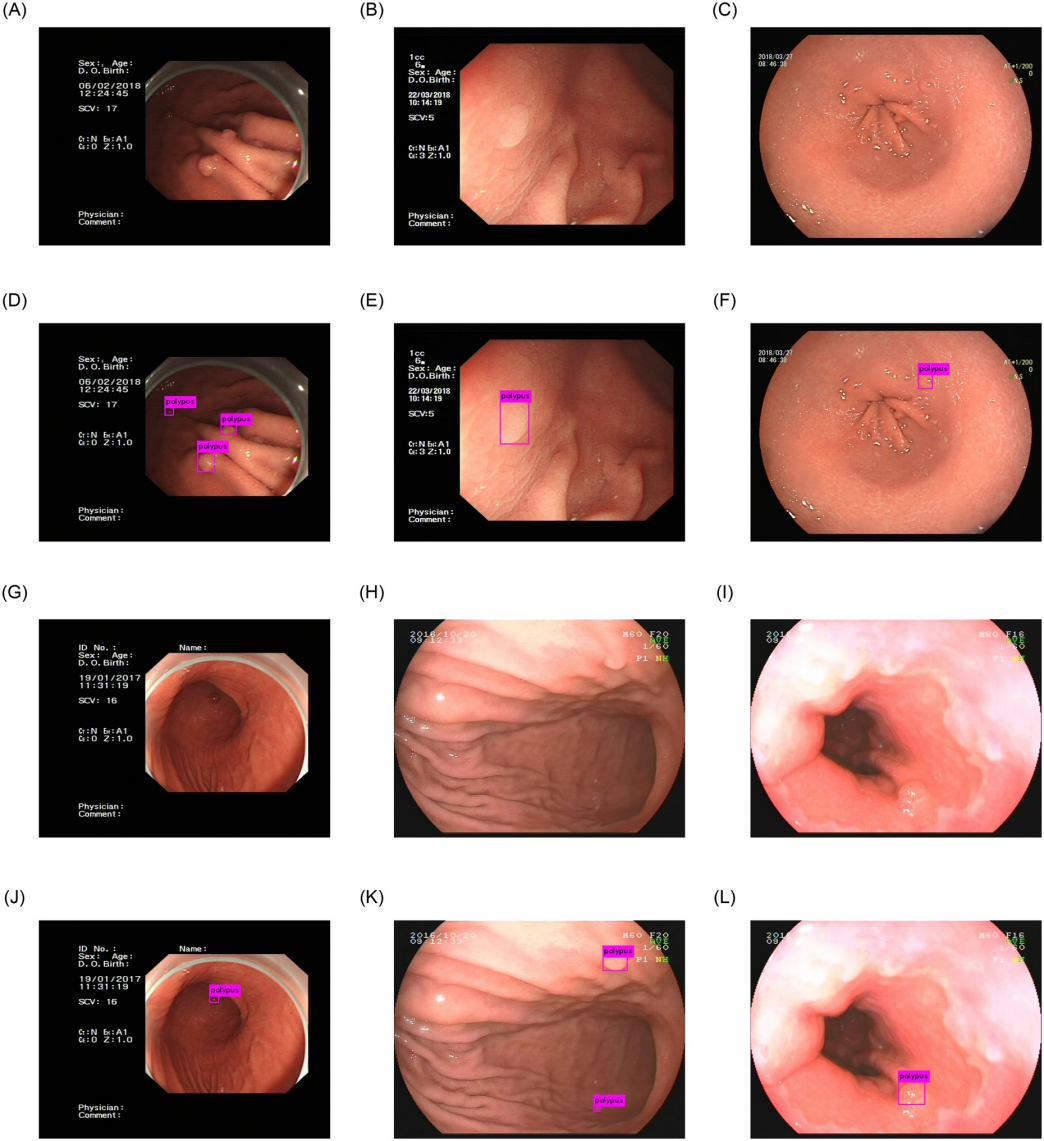
The results of our network. Fig(A), (B), (C), (G), (H), (I) are the origin images of gastric polyps. Fig(D), (E), (F), (J), (K), (L) are the detection results of gastric polyps.

In conclusion, compared to other polyps detection works, our work has achieved good results in the detection of gastric polyps on our dataset. Fig 9 shows some examples of gastric polyps detection using our method. This detection network performs well in the presence of a large number of small gastric polyps in an image. But there are still some deficiencies in the detection of gastric polyps with very obscure features. Effectively improving the accuracy of gastric polyps detection and feature extraction ability of our network is the main research direction in the future.

## 6. Conclusion

In this paper, we propose a feature extraction and fusion module to improve the detection ability of small gastric polyps and feature extraction ability. We combine this module with the YOLOv3 network. Through the experimental evaluation, our network exhibits the potentials for detecting gastric polyps. In each evaluation metrics, our method has yielded good results than other previous methods. The high polyps detection performance shows that our method can reduce the risk of misdetection and missed detection in gastroscopy examination. Our work will be useful to assist doctors in detecting and diagnosing gastric polyps. In the future, we intend to integrate our method with a classification network to judge whether a polyp is adenomatous or proliferative. And we will improve our network structure by applying new CNN architecture and feature module to achieve better performance.
